# Evaluation of the humoral immune responses in adult cattle and sheep, 4 and 2.5 years post-vaccination with a bluetongue serotype 8 inactivated vaccine^☆^

**DOI:** 10.1016/j.vaccine.2013.06.033

**Published:** 2013-08-20

**Authors:** C.A. Batten, L. Edwards, C.A.L. Oura

**Affiliations:** a The Pirbright Institute, Ash Road, Woking, Surrey, GU24 0NF, UK; b School of Veterinary Medicine, University of the West Indies, Trinidad and Tobago

**Keywords:** Bluetongue, Inactivated vaccines, Humoral response, Protection

## Abstract

One of the big surprises about the devastating outbreak of bluetongue serotype-8 that spread across Northern and Western Europe between 2006 and 2008 was how relatively quickly the virus was controlled and eradicated from affected countries. This was at least in part attributed to the high levels of vaccine coverage achieved in affected countries. A previous study revealed that neutralising antibodies persisted in the majority of vaccinated cattle for at least 3 years post-vaccination, indicating that cattle are likely to be protected for this time period. The current study revealed that neutralising antibodies persisted in the same group of cattle for up to 4 years post-vaccination, and that neutralising antibodies persisted for up to 2.5 years in sheep that had been vaccinated on two occasions one year apart. These results have implications for future bluetongue surveillance programmes and vaccine control strategies.

## Highlights

• ELISA antibodies persist in serum and milk for at least 4 years post-vaccination in cattle.
• ELISA-based milk/serum surveillance is not possible for at least 4 years post-vaccination in cattle, but surveillance is possible in young unvaccinated cattle.
• Neutralising antibodies persist for at least 4 years post-vaccination (two vaccine doses four weeks apart) in cattle.
• Neutralising antibodies persist for at least 2.5 years post-vaccination (two vaccine doses one year apart) in sheep.
• Protection is likely for at least 4 and 2.5 years post-vaccination in cattle and in sheep respectively.

## 1 Introduction

The vaccination campaigns, using inactivated vaccines against bluetongue serotype-8 (BTV-8), were highly successful in controlling and eradicating BTV-8 from affected countries in Northern and Western Europe. This was despite some countries using voluntary vaccination campaigns and other countries reverting from compulsory to voluntary vaccine campaigns after one or two years, which resulted in some farmers choosing not to revaccinate their livestock in the second and third years of the outbreak [1]. The reduced vaccine coverage that ensued resulted in experts fearing a resurgence of the virus. However, despite the reduced levels of vaccine coverage in some countries in the second and third year of the outbreak, the amount of cases of BTV-8 reported across Europe continued to decrease dramatically, with no cases of BTV-8 reported in Northern and Western Europe in 2010. By 2012 most countries in Northern and Western Europe had successfully eradicated BTV-8 from their territories [2].

The commercially available BTV-8 inactivated vaccines have shown good safety and efficacy in both cattle and sheep, and studies have shown that both sheep and cattle are protected for at least 1 year post-vaccination [3–8]. A recent study revealed that group-specific antibodies persisted at high levels in milk and serum in a high percentage of cattle for at least 3 years post-vaccination, thus removing the option of using these animals in ELISA-based surveillance programmes. The same study showed that neutralising antibodies persisted in a high percentage of cattle for at least 3 years post-vaccination, indicating that cattle are likely to be protected for this time period [9].

The objectives of this study were to follow up on the previous study [9] and to assess the status of the humoral immune response in the same group of adult cattle 4 years after the initial course of BTV-8 vaccination. The study also assessed the status of the humoral immune response in a group of adult sheep 2.5 years after a second BTV-8 vaccination, given 1 year after the primary vaccination. This will provide information on the length of persistence of antibodies post-vaccination in the blood and milk for surveillance purposes, as well as the persistence of neutralising antibodies, which is likely to be linked to protection.

## 2 Materials and methods

### 2.1 Cattle and experimental design

Twenty-nine adult Friesian–Holstein cattle from a dairy farm in the county of Surrey, UK, that had been previously sampled and tested for antibodies to BTV in both milk and serum [9], were selected for resampling in this study. These cattle were considered not to have been naturally infected with BTV-8 during the BTV-8 outbreak in the UK in 2007 due to the fact that no clinical cases of BT were confirmed in the county of Surrey throughout 2007, and no further cases of BTV-8 were declared in the UK between 2008 and 2011 [9]. The cattle were vaccinated by sub-cutaneous injection in the neck region on two occasions, 4 weeks apart (according to the manufacturer's instructions) in May and June 2008 with the Intervet-manufactured Bovilis-BTV-8 (Intervet, Germany) inactivated vaccine. The cattle were not revaccinated between 2009 and 2012.

The 29 cattle were sampled in June 2012. Whole blood (serum) samples and individual milk samples were collected, unfortunately a milk sample was not collected from one animal. These 29 cattle had been previously tested for the presence of BTV antibodies in 2011 [9].

### 2.2 Sheep and experimental design

Twenty-three adult sheep of mixed breeds from a farm in Surrey, UK were selected for the study. The sheep were vaccinated by sub-cutaneous injection in the neck region with a single dose (according to the manufacturer's instructions) on two occasions in May 2008 and again in May 2009 with the Intervet-manufactured Bovilis-BTV-8 (Intervet, Germany) inactivated vaccine. The sheep were not revaccinated between 2010 and December 2011. Whole blood (serum) samples were collected from the 23 sheep in December 2011.

### 2.3 Serology

#### 2.3.1 ELISA

The detection of BTV specific antibodies in serum was carried out using a sandwich (double antigen) ELISA (sELISA, ID-Screen Bluetongue Early detection ELISA, ID-Vet, France) according to the manufacturer's instructions. The detection of BTV specific antibodies in milk was carried out using the ID Screen® Bluetongue Milk ELISA (ID-Vet, France) according to the manufacturer's instructions.

#### 2.3.2 Serum neutralisation test (SNT)

SNT was performed according to the method of Haig [10] using the BTV-8 South African reference virus and serotype-specific BTV-8 positive control antisera [9].

## 3 Results

### 3.1 Cattle 4 years post vaccination

A group of 29 adult dairy cattle, that had been given two shots of inactivated BTV-8 vaccine 4 weeks apart in May/June 2008 and had not been revaccinated since, were sampled 4 years after the primary course of vaccination in June 2012. Serum samples were tested for the presence of group specific BTV antibodies by a sELISA and for the presence of type-specific neutralising antibodies to BTV-8 by SNT. Individual milk samples were tested for the presence of group-specific BTV antibodies by ELISA. From the 29 serum samples, 28 (97%) were positive in the sELISA and SNT, with neutralising titres ranging from log_10_ 1–2.38. Twenty-three of the 28 individual milk samples (82%) were positive for BTV antibodies in ELISA (Table 1).

### 3.2 Sheep 2.5 years post vaccination

A group of 23 adult sheep, that had been vaccinated with inactivated BTV-8 vaccine in May 2008 and 2009 and had not been revaccinated since, were sampled after 2.5 years in December 2011. Serum samples were tested for the presence of group specific BTV antibodies by a sELISA and for the presence of type-specific neutralising antibodies to BTV-8 by SNT. From the 23 serum samples, 23 (100%) were positive in the sELISA and SNT, with neutralising titres ranging from log_10_ 1.30–2.38 (Table 2).

## 4 Discussion

One of the big surprises related to the devastating outbreak of BTV-8 that spread across Northern and Western Europe between 2006 and 2008, was how relatively quickly the virus was controlled and eradicated from affected countries. The principal contributing factor was likely to be related to the high levels of natural and vaccine-related immunity present in the susceptible ruminant populations throughout the affected regions. In many European countries that were affected by the virus, a high percentage of susceptible ruminants were naturally infected during the outbreak. A recent study showed that high levels of neutralising antibodies were induced in naturally infected cattle for four to six years after the last exposure to BTV-8, indicating that naturally infected animals are likely to remain protected for many years [11].

A key question that remained unaddressed was for how long cattle and sheep were likely to be protected for after vaccination with the inactivated BTV-8 vaccines on the market. A previous study [9], revealed that neutralising antibodies persisted in the majority of vaccinated cattle for at least 3 years post-vaccination, indicating that the cattle are likely to be protected for this time period. The same authors have now revealed that neutralising antibodies persisted in the same group of cattle for up to 4 years post-vaccination, and that neutralising antibodies persisted for up to 2.5 years in sheep that had been vaccinated on two occasions one year apart. Neutralising antibodies are known to play a key role in protecting animals from disease and viraemia and there is known to be a strong correlation between the presence of neutralising antibodies and protection [4,12–14]. These results therefore indicate that cattle are likely to be protected for at least 4 years after the initial course of two vaccines, and that sheep, provided they are vaccinated on two occasions one year apart, are likely be protected for at least 2.5 years after the second vaccination. There is however a need to confirm this protection through challenge studies.

These results have implications for future bluetongue surveillance and control programmes in that group-specific antibodies in both individual and bulk milk samples, as well as serum samples, persist in the majority of vaccinated cattle for at least 4 years, and in sheep for at least 2.5 years, thus precluding the use of these tests for surveillance purposes during this time period. It would however be possible to carry out surveillance using younger cattle/sheep that had not been vaccinated. The results also reveal that BTV inactivated vaccines may produce far longer periods of immunity in cattle and sheep than originally thought, which may have contributed to the rapid and efficient eradication of BTV-8 from Northern and Western Europe, despite reduced vaccination coverage in the 2nd and 3rd year of the outbreak.

## Figures and Tables

**Table 1:** Persistence of antibodies, measured by ELISA and SNT, in the milk and blood of adult dairy cattle four years post-vaccination with a BTV-8 inactivated vaccine.

Cow number	Milk ELISA (%S/P)^a^		Early detection ELISA (%S/P)^b^		SNT log_10_	
	2011	2012	2011	2012	2011	2012
1	Pos (290)	Pos (153)	Pos (184)	Pos (254)	Pos (1.18)	Pos (1.48)
2	Pos (221)	**Neg (79)**	Pos (184)	Pos (251)	Pos (1.48)	Pos (1.00)
3	Pos (262)	Pos (142)	Pos (165)	Pos (267)	Pos (1.78)	Pos (1.78)
4	**Neg (68)**	**Neg (24)**	Pos (178)	Pos (224)	Pos (1.60)	Pos (1.48)
5	Pos (307)	No sample	Pos (193)	Pos (280)	Pos (1.00)	Pos (1.78)
6	Pos (305)	Pos (144)	Pos (190)	Pos (255)	Pos (2.38)	Pos (2.20)
7	Pos (206)	Pos (134)	Pos (193)	Pos (267)	Pos (1.60)	Pos (2.20)
8	Pos (140)	Pos (113)	Pos (193)	Pos (280)	Pos (2.20)	Pos (1.78)
9	Pos (301)	Pos (145)	Pos (183)	Pos (262)	Pos (1.78)	Pos (1.78)
10	Pos (295)	Pos (146)	Pos (192)	Pos (255)	Pos (2.08)	Pos (2.38)
11	Pos (292)	Pos (146)	Pos (122)	Pos (185)	Pos (1.60)	Pos (1.90)
12	Pos (189)	Pos (125)	Pos (153)	Pos (230)	Pos (1.18)	Pos (1.60)
13	Pos (281)	Pos (131)	Pos (193)	Pos (280)	Pos (1.48)	Pos (1.18)
14	Pos (286)	Pos (139)	Pos (170)	Pos (240)	Pos (1.18)	Pos (1.60)
15	Pos (229)	Pos (125)	Pos (183)	Pos (264)	Pos (1.18)	Pos (1.00)
16	Pos (298)	Pos (152)	Pos (187)	Pos (266)	Pos (1.78)	Pos (2.08)
17	Pos (304)	Pos (148)	Pos (193)	Pos (266)	Pos (1.30)	Pos (1.78)
18	Pos (300)	Pos (137)	Pos (185)	Pos (268)	Pos (2.51)	Pos (1.90)
19	Inc (98)	**Neg (68)**	Pos (165)	Pos (198)	Pos (1.00)	Pos (1.18)
20	Pos (241)	Pos (141)	Pos (193)	Pos (276)	Pos (1.30)	Pos (1.48)
21	Pos (247)	Pos (124)	Pos (191)	Pos (262)	Pos (1.48)	Pos (1.18)
22	Pos (260)	Pos (114)	Pos (193)	Pos (240)	Pos (1.60)	Pos (1.60)
23	Pos (306)	Pos (150)	Pos (193)	Pos (270)	Pos (1.78)	Pos (1.78)
24	Pos (306)	Pos (153)	Pos (189)	Pos (268)	Pos (1.00)	Pos (1.18)
25	Pos (301)	Pos (151)	Pos (187)	Pos (269)	Pos (1.48)	Pos (1.60)
26	**Neg (7)**	**Neg (-8)**	**Neg (22)**	**Neg (18)**	**Neg**	**Neg**
27	**Neg (52)**	Pos (135)	Pos (176)	Pos (263)	Pos (1.18)	Pos (1.18)
28	**Neg (50)**	**Neg (58)**	Pos (107)	Pos (99)	Pos (1.30)	Pos (1.78)
29	Pos (268)	Pos (137)	Pos (181)	Pos (248)	Pos (1.60)	Pos (1.60)

SNT: serum neutralisation test; Pos: positive; Neg: negative; Inc: inconclusive. % S/P: percentage of positivity compared to the positive control. Bold value shows the negative results.

a Individual samples: negative ≤90%, inconclusive 90–110%, positive ≥110%.

b Negative ≤25%, inconclusive 25–30%, positive ≥30%.

**Table 2:** Persistence of antibodies, measured by ELISA and SNT, in the blood of sheep 2.5 years post-vaccination (2 vaccines given one year apart) with a BTV-8 inactivated vaccine.

Animal number	Early detection ELISA (%S/P)^a^	SNT log_10_
1	Pos (242)	Pos (1.90)
2	Pos (256)	Pos (1.78)
3	Pos (231)	Pos (1.48)
4	Pos (256)	Pos (1.90)
5	Pos (231)	Pos (1.48)
6	Pos (249)	Pos (2.20)
7	Pos (261)	Pos (1.60)
8	Pos (241)	Pos (2.08)
9	Pos (242)	Pos (2.08)
10	Pos (244)	Pos (1.60)
11	Pos (181)	Pos (1.78)
12	Pos (253)	Pos (1.90)
13	Pos (194)	Pos (2.08)
14	Pos (251)	Pos (1.30)
15	Pos (239)	Pos (1.60)
16	Pos (247)	Pos (1.60)
17	Pos (252)	Pos (2.38)
18	Pos (254)	Pos (2.20)
19	Pos (229)	Pos (1.60)
20	Pos (268)	Pos (2.08)
21	Pos (232)	Pos (2.08)
22	Pos (216)	Pos (2.38)
23	Pos (231)	Pos (1.78)

SNT: serum neutralisation test; Pos: positive; Neg: negative; Inc: inconclusive. % S/P: percentage of positivity compared to the positive control.

a Negative ≤25%, inconclusive 25–30%, positive ≥30%.
